# Using ethnographic approaches to document, evaluate, and facilitate virtual community-engaged implementation research

**DOI:** 10.1186/s12889-023-15299-2

**Published:** 2023-02-28

**Authors:** Borsika A. Rabin, Kelli L. Cain, Linda Salgin, Paul L. Watson, William Oswald, Bonnie N. Kaiser, Lawrence Ayers, Crystal Yi, Alexander Alegre, Jessica Ni, Allyn Reyes, Kasey E. Yu, Shelia L. Broyles, Robert Tukey, Louise C. Laurent, Nicole A. Stadnick

**Affiliations:** 1 Herbert Wertheim School of Public Health and Human Longevity Science, University of California San Diego, La Jolla, San Diego, CA United States of America; 2 UC San Diego Altman Clinical and Translational Research Institute Dissemination and Implementation Science Center, University of California San Diego, La Jolla, San Diego, CA United States of America; 3 San Ysidro Health, San Diego, CA United States of America; 4 Joint Doctoral Program in Public Health, San Diego State University, University of California San Diego, San Diego, United States of America; 5 The Global Action Research Center, San Diego, CA United States of America; 6 Department of Anthropology and Global Health Program, University of California San Diego, La Jolla, San Diego, CA United States of America; 7 Department of Obstetrics, Gynecology, and Reproductive Sciences, University of California San Diego, La Jolla, San Diego, CA United States of America; 8 Department of Urban Studies and Planning, University of California San Diego, La Jolla, San Diego, CA United States of America; 9 Department of Pediatrics, University of California San Diego, La Jolla, San Diego, CA United States of America; 10 UC San Diego Altman Clinical and Translational Research Institute Community Research Partnerships, University of California San Diego, La Jolla, San Diego, CA United States of America; 11 Laboratory of Environmental Toxicology, Department of Pharmacology, University of California San Diego, La Jolla, San Diego, CA United States of America; 12 Department of Psychiatry, University of California San Diego, La Jolla, San Diego, CA United States of America; 13 Child and Adolescent Services Research Center, San Diego, CA United States of America

**Keywords:** Community engagement, Ethnographic methods, Qualitative methods, Implementation science, Health equity, COVID-19

## Abstract

**Background:**

Community Advisory Boards (CABs) have been frequently used to engage diverse partners to inform research projects. Yet, evaluating the quality of engagement has not been routine. We describe a multi-method ethnographic approach documenting and assessing partner engagement in two “virtual” CABs, for which we conducted all meetings remotely.

**Methods:**

Two research projects for increasing equitable COVID-19 testing, vaccination, and clinical trial participation for underserved communities involved remote CAB meetings. Thirty-three partners representing 17 community groups participated in 15 sessions across the two CABs facilitated by a social change organization. We developed ethnographic documentation forms to assess multiple aspects of CAB member engagement (e.g., time spent speaking, modality used, types of interactions). Documenters were trained to observe CAB sub-groups via virtual sessions. Debriefing with the documentation team after CAB meetings supported quality assurance and process refinement. CAB members completed a brief validated survey after each meeting to assess the quality and frequency of engagement. Content and rapid thematic analysis were used to analyze documentation data. Quantitative data were summarized as frequencies and means. Qualitative and quantitative findings were triangulated.

**Results:**

A total of 4,540 interactions were identified across 15 meetings. The most frequent interaction was providing information (44%), followed by responding (37–38%). The quality and frequency of stakeholder engagement were rated favorably (average 4.7 of 5). Most CAB members (96%) reported good/excellent engagement. Specific comments included appreciation for the diversity of perspectives represented by the CAB members and suggestions for improved live interpretation. Debriefing sessions led to several methodological refinements for the documentation process and forms.

**Conclusion:**

We highlight key strategies for documenting and assessing community engagement. Our methods allowed for rich ethnographic data collection that refined our work with community partners. We recommend ongoing trainings, including debriefing sessions and routinely reviewed assessment of data to strengthen meaningful community engagement.

**Supplementary Information:**

The online version contains supplementary material available at 10.1186/s12889-023-15299-2.

## Background

Consistent and meaningful engagement of diverse partners in research has been identified as a priority and method to increase the relevance and impact of scientific outputs for end beneficiaries [1–3]. Partner engagement is especially critical when addressing the priorities and needs of underserved communities who experience a disproportionate burden of morbidity and mortality from health conditions and social injustice and whose voices have historically not been well-integrated into research. Community engagement is at the heart of the conceptual model focused on health equity through transformed systems for health with ‘strengthened partnerships’ identified as a domain of measurable outcomes [4] (Organizing Committee for Assessing Meaningful Community Engagement in Health & Health Care Programs & Policies). Meaningful community engagement throughout phases of design, implementation, adaptation, and evaluation provides a critical, evidence-based approach to addressing public health challenges [5, 6]. Implementation of health interventions have greater reach and impact when the focus is on interweaving health promotion strategies, practices, programs, and policies to fit within or enhance existing settings and environmental contexts rather than focusing solely on individual behavior changes [7]. These principles are at the heart of implementation science, community-based participatory action research, and public health. Community Advisory Boards (CABs) serve as ongoing partnerships to address community health concerns and a mechanism for building capacity in the community and the academic institution [8]. Brockman et al. [9] reported that their CAB was helpful in generating/refining ideas, identifying community partners, culturally tailored and targeted recruitment strategies, intervention design and delivery and dissemination. The roles of CABs in partner-engaged research needs further exploration.

The COVID-19 pandemic dramatically illustrates widening health disparities impacting immigrant, refugee, and Black, Indigenous, and People of Color communities nationally in the United States and within specific geographic regions [10]. These communities are significantly more likely to experience mortality and morbidity from COVID-19, along with delayed and lower testing and vaccination rates compared to white individuals in the United States [11]. Drivers of these disparities are multifactorial, multi-level, and often intersecting with cumulative and compounding impacts [12, 13]. To advance health equity in COVID-19 testing and vaccination access and uptake, public health interventions must have high acceptability, usability, and uptake by end-beneficiaries and should fit with the policy and organizational infrastructures. To create solutions that meet these criteria, members of underserved communities must be included in health research design, conduct, and evaluation.

Community Advisory Boards (CABs) have been frequently used to engage diverse partners, including members of underserved communities, to inform research projects. How CABs are operationalized – who they include, how often they meet, what they use for content and format, and how they are evaluated – greatly varies across projects and is not well documented. How suggestions generated by CABs are utilized in project design and decision-making is also not well documented.

An added complexity when working with CABs whose members are from underserved communities is the need to consider language preferences, which can necessitate translation and interpretation services before, during, and after CAB meetings to allow for full CAB engagement. Furthermore, the COVID-19 pandemic forced social gatherings, including CAB interactions, to embrace convening on virtual formats. These virtual formats highlighted unique challenges for community-engaged research activities and further underscored the need to understand how much and how well engagement happens during CAB meetings [14].

Evaluation of the quality and degree of engagement via CABs has not been routinely conducted and/or reported [15–18]. Moreover, most methods for the assessment of partner engagement are narrow in scope, rarely use a multi- or mixed-method approach, and are not easily replicated across context and studies [18]. A comprehensive synthesis of frameworks to support the engagement of community members in research identified over 60 frameworks. Despite their quantity, these frameworks had low usage and rarely linked to methods or measures for assessing breadth or depth of engagement [19].

Ethnographic approaches are increasingly used in implementation science, including for evaluation of engagement of partners, because they are well suited to provide a contextual understanding of processes, complex interactions, and diverse views from stakeholders [20]. Recommendations for the use of ethnographic approaches in implementation science include iterative development of methodologies, valuing the reflexivity of the researcher/documenter, and contextualizing findings through considering the local and broader context and perspectives from stakeholders at multiple levels. In our work, we used ethnographic methods to document the quality and degree of CAB member engagement within and across CAB sessions. Our objective is to describe our multi-method ethnographic approach to documenting and assessing engagement. We applied the approach in two virtual CABs engaging underserved communities in implementation science projects funded through National Institutes of Health (NIH) research initiatives to eliminate disparities in COVID-19 testing and clinical trial participation, access to care, and vaccine uptake.

## Methods

### Study context

#### COVID-19 implementation projects

There were two projects funded through the NIH Rapid Acceleration of Diagnostics-Underserved Populations (RADx-UP) [21] and Community Engagement Alliance (CEAL) Against COVID-19 Disparities [22] initiatives aimed at promoting equitable access to COVID-19 testing, vaccination, and inclusion in clinical trials where our documentation and assessment of partner engagement was undertaken. The MASKED Institutional Review Boards approved both studies.

Community-driven optimization of COVID-19 testing to Reach and Engage Underserved Areas for Testing Equity (CO-CREATE) CO-CREATE is funded through the NIH RADx-UP initiative to understand practices, barriers, and facilitators to access and uptake of COVID-19 testing and follow-up for underserved community members from the perspectives of patients, providers, and organizational leaders at a federally qualified health center near the U.S./Mexico border.

Share, Trust, Organize, Partner: the COVID-19 California Alliance (STOP COVID-19 CA) The STOP COVID-19 CA project is funded by the NIH CEAL program that includes community-academic teams in 11 states throughout the U.S. and focuses on COVID-19 awareness and education research, especially among Black, Latino, Indigenous, refugee, and immigrant populations. The California CEAL team is locally known as STOP COVID-19-CA and involves a network of 11 California institutions, including UC San Diego. Our aim was to conduct a rapid community engagement project to assess multi-level barriers, facilitators, and processes to engaging individuals from underserved communities, particularly Black, Indigenous, and People of Color and African, Asian, and Middle Eastern immigrant and refugee communities, in COVID-19 clinical trials, as well as to advance vaccine uptake.

#### Community Advisory boards

The CO-CREATE and UC San Diego STOP COVID-19 CA projects involved parallel virtual CAB meetings that engaged in Theory of Change and Appreciative Inquiry processes. Thirty-three stakeholders representing 17 community groups across the two CABs participated in 15 sessions from November 2020 to April 2021 to inform the co-creation of testing and vaccine equity strategies. Identification and recruitment of CAB members for both projects were led by the Global Action Research Center (ARC), a non-profit social change organization with expertise conducting participatory action research to address public health and environmental justice needs. The Global ARC has strong community leadership and advocacy ties within the broader San Diego area and is considered a trusted resource to local grassroots organizations and policymakers. The Global ARC was in an excellent position to engage key members of the community, considering the target communities’ familiarity with Global ARC, knowledge of community culture, and existing formal and informal community leadership [23] The leadership of the Global ARC has worked with these communities for over twenty-five years, building deep, trusting relationships.

The composition of the CABs differed across the two projects and reflected the specific focus and goals of each project. However, an overarching guide for the selection of members for both CABs was based on the individual being able to represent their community and bring their community’s voices forward rather than being a representative of their community. This is a critical principal of meaningful community engagement. Someone who represents their community differs from someone who is representative of their community in that their ability to bring their community’s voice forward is because they are connected and accountable to that community. Someone who is representative of a community, while sharing a set of experiences with others who are demographically similar, only speaks for themselves. Without accountability there is no representation and no guarantee that the representative person shares the views of their community.”

People were invited to be part of these advisory boards because of their role in their communities. Each of these individuals are well-known and respected members of their community who are looked to for knowledge, information, guidance, and advice. Invitees included Promotores and key leaders within several cultural/linguistic communities who not only bring knowledge and information to their community but also bring the community’s perspective, issues, and concerns back to the advisory boards. Selecting, inviting, and building support networks for the CAB members took the most amount of person hours in the startup phase [24]. A native Spanish-speaker and employee of Global ARC served as the direct liaison with our Spanish-speaking community members on the CABs. CAB members were provided $100 stipends for their participation in each meeting for their time participating and sharing their expertise in the CAB meetings. The Global ARC mailed stipends in the form of gift cards to CAB meeting attendees and confirmed receipt by email, text, or phone calls. Previously published papers on the co-creation of the Theory of Change [23] and the community engagement resources needs and costs associated with engaging underserved communities [24] include more details about CAB processes. Table 1 provides a summary of the CAB members for each project.

**Table 1:** CAB Partners for CO-CREATE and UC San Diego STOP COVID-19 CA

CO-CREATE	STOP COVID-19 CA
**9 Community Partners** • Promotores Coalition* • Latinos y Latinas en Acción*	**11 Community Partners** • Comite Organizador Latino de City Heights* • Karen Organization of San Diego • Kupanda Kids • Partnership for the Advancement of New Americans • Refugee Health Unit/Center for Community Health • Somali Bantu Community • South Sudanese Community Center • The Humanity Movement • Unity in the Community • Youth Will
**6 Public Health Research Partners** • University of California San Diego • San Diego State University • Loma Linda University	**2 Policy Partners (non-voting CAB members)** • San Diego City Council, District 9, Community Empowerment
**7 Health Clinic Partners** • Providers • Administrators	

*Spanish-speaking members. Live Spanish/English interpretation and translation occurred during CAB meetings.

The CO-CREATE CAB included 22 members who identified as community residents (i.e., Community Partners), public health researchers (i.e., Public Health Research Partners), and clinical partners (i.e., Health Clinic Partners). The goal of the CO-CREATE CAB was to directly inform co-creation of implementation strategies for a tailored COVID-19 testing program that is currently being implemented in a federally qualified health center. The STOP COVID-19 CA CAB was composed of 11 community leaders from diverse communities from 10 local grassroots community organizations (i.e., Community Partners) and two policymakers (i.e., Policy Partners), and the goal was to inform materials and resources needed to support vaccine clinical trial participation and equity initiatives in underserved communities. In this CAB, we identified key Cultural weavers within their communities. All of these individuals spoke English, which facilitated their skills and expertise as Weavers, so they were able to meaningfully participate in the discussion in English. Across both CABs, we had members who preferred Spanish as their primary language and we offered live Spanish-to-English translation and interpretation to these members.

CAB meetings were conducted virtually using the Zoom and Miro interactive online platforms. Technology devices and assistance were provided to CAB members to ensure equitable participation in virtual meetings. Meetings were scheduled for two hours at least once a month in the late afternoon/early evening and were facilitated by the Global ARC. Live Spanish interpretation and written translation of materials were provided, and sessions were video recorded. Structured documentation forms were used by a team of trained documenters to capture observable data about engagement practices (e.g., time each CAB member spent speaking).

A total of seven sessions were conducted for each project to complete the Theory of Change process, a comprehensive description and illustration of how and why a desired change is expected to happen in a particular context. One Appreciative Inquiry session for CO-CREATE was also completed and documented. Appreciative Inquiry sessions involve a process to assess whether progress is made with the implementation of necessary conditions and indicators of success identified during the Theory of Change process. Sessions used a combination of large group and small group activities using the breakout room function of the Zoom platform. More details about the specific content of the sessions have been published elsewhere [23].

### Ethnographic Documentation and Assessment

We used a multi-method approach to documenting and assessing the quality and extent of member engagement across the two virtual CABs. Methods included (1) documentation of CAB meeting processes, (2) a post-session survey of CAB members, the research team, and community partners on engagement, (3) periodic reflections and debriefing sessions between the research team and the Global ARC to discuss CAB processes and content.

#### Ethnographic and qualitative documentation of CAB meeting processes

Ethnographic documentation forms were adapted from a form previously used by the Global ARC based on literature review and guidance from an ethnographer on our research team (BK) and our community partners at the Global ARC (PW, WO). The form was further refined iteratively through pilot testing and debriefing meetings. Led by community partners, we decided to use the less “research-centric” term “innovation documentation” for the process and form instead of “observation” to avoid potential concerns from CAB members about being observed and to highlight the relatively novel approach to assessing engagement. The term ‘observation’ has a strong, negative connotation for historically marginalized and underrepresented communities, while the term “*innovation* documentation” highlights the critically important yet often overlooked aspects of community engagement. During the first CAB meetings, we provided a detailed description of the intent and process of this data collection, introduced our innovation documentation team, and allowed for questions to be asked about the process. Specifically, we explained that we were assessing community engagement using a combination of structured and more open documentation looking at (1) a quantitative survey of the quality and quantity of engagement and (2) innovation documentation notes. We explained that we would record the CAB sessions with permission of the CAB members to add more details to the engagement documentation notes. We showed the CAB members the engagement survey items and explained it would be sent to all meeting attendees after each session with a voluntary invitation to share their confidential experiences of engagement during each meeting. During the detailed description of the process, we also indicated the dual role of this data collection: (a) ongoing improvement of our approaches to better engage with our partners; and (b) use of these data to describe our novel approach to the engagement process since detailed descriptions of ongoing, meaningful community engagement are rare (hence the term ‘innovation’).

The documentation form allowed us to gather information on various aspects of CAB members’ participation, including attendance, time spent speaking, primary language (English/Spanish), modality used (computer/phone/both), arrival and departure time, and interruptions (i.e., who interrupted whom and reason for interruption). Documenters also identified each CAB meeting participant (including members and non-voting members) as having one or more of the following roles: no active role, provided input, identified priorities, participated in program design, set the agenda, and/or led or co-led the meeting. Documenters provided open-ended comments about each CAB member noting any additional observations (e.g., technology challenges). The form had a dedicated section to document the type and content of interactions during the meeting. An interaction was defined as an individual making a statement, asking or answering a question, or providing a general comment or summation to either another individual or a group of people during the meeting. For every interaction, the sender was identified by name and the target audience was identified as either an individual, a subgroup, or the entire group. Each time a new individual spoke, a new interaction was created. Information was also collected on the content of the interaction, the type of interaction (seeking information, giving information, response, summation, or other), and open-ended comments for any additional observations. Documenters were trained on these methods and debriefings after each CAB meeting allowed for opportunities for documenters to ask questions as their forms were reviewed.

The documentation team included nine academic team members: seven undergraduate students and two Master’s-level research team members. Documenters participated in an initial 2-hour interactive training by an ethnographer (BK), implementation scientists (NS, BR), and community engagement experts (PW, WO). In addition, documenters participated in a 1-hour debrief meeting following each CAB session to review their documentation forms and refine CAB practices.

To facilitate focused documentation of information, documenters were assigned to a specific CAB sub-group (e.g., Community Partners, Public Health Research Partners, Health Clinic Partners). A rotating schedule was implemented to reduce potential bias in documenters observing the same sub-group for each meeting. Each documenter was also assigned a section of the documentation form to promote high quality data collection. A combination of live and recorded meetings was used to complete the documentation forms.

#### Analysis of documentation data

Data from multiple documenters and sessions were compiled by the lead analyst (KC). We used a combination of quantitative and qualitative analytic approaches to analyze data from the documentation forms. Content analysis on close-ended data and rapid thematic analysis on open-ended data were conducted to summarize quantitative and qualitative data, respectively, from the documentation forms.

Analysis and summary of quantitative data from the documentation forms The following data were extracted from the documentation forms for quantitative analysis: attendees, time spent speaking (i.e., minutes), primary language (i.e., English or Spanish), modality used (i.e., computer, phone, or both), arrival and departure time, sender and target for each interaction, and types of stakeholder interactions (e.g., seeking information, giving information). For analysis on the senders of information, individuals were categorized by the group they represented (e.g., community partner) and counts were generated as the number of each type of interaction (e.g., giving information) for each sender group and each target group. Descriptive statistics were calculated for each variable.

Roles of CAB members during the meetings were selected from a list of possible roles that included: no active role, provided input, identified priorities, participated in program design, set the agenda, and led or co-led the meeting. To be counted as serving in a role, at least one documenter needed to endorse the role. All participating documenters completed the roles survey for each meeting and responses were averaged across meetings for each project.

Analysis and summary of qualitative data from the documentation forms Qualitative data included comments from the documenters about individual CAB members and content of interactions during CAB meetings. We used a rapid thematic analysis approach to identify overarching themes for these sections. Initial review of qualitative data resulted in a preliminary set of themes that were reviewed and agreed upon by the research team. When new themes emerged during the coding process, they were noted by the analyst and reviewed by the larger team. All content was double coded by two Master’s level analysts (LA, LS), and the lead analyst (KC) resolved differences between coders.

Refinement of documentation forms At the conclusion of our documentation process, we surveyed documenters and the research team on the usefulness of sections in the documentation form. The lead analyst (KC) compiled the information and modifications to the documentation form were proposed. The research team reviewed the proposed changes and the revised form was finalized. This process allowed for a refined and simplified documentation form for future projects (available as Additional File 1).

#### Post-session survey of CAB members, the research team, and community partners on engagement

After each CAB meeting, all attendees were invited to complete a brief online survey based on a validated survey of stakeholder engagement by Goodman and colleagues [25]. The survey included nine items and was intended to assess the quality (“How *well* do the partners leading the research do each of the following?”) and the frequency (“How *often* do the partners leading the research do each of the following?”) of various aspects of engagement. Response options for items assessing quality ranged from poor to excellent. Response options for items assessing frequency ranged from never to always. An optional open-ended comment field allowed for the sharing of any observations, comments, or suggestions related to the most recent CAB meeting.

Analysis of CAB member engagement survey data After each CAB meeting, summarized survey responses and open-ended comments were reviewed during debriefing sessions to inform refinements to the CAB process. During the main analysis, survey findings were reviewed for patterns over time.

#### Periodic reflections and debriefing sessions

After each CAB meeting, we held two debriefing sessions. The first debriefing session was held with the research team and Global ARC for 15–30 min immediately following CAB meetings to informally discuss how the session went, concerns we detected, and potential changes we needed to make to improve engagement for future sessions. These debriefing sessions included a Spanish-speaking employee of the Global ARC who was the direct liaison to the Spanish-speaking CAB community members.

In addition, the research team, documenters, and the Global ARC met for 1-hour formal periodic reflections and debriefing sessions a few days after each meeting. Periodic reflections are common in ethnographic methods, including using guided discussions to document events and diverse viewpoints throughout the implementation of a project [26]. In addition to reflections from each member of the team, CAB processes, content, CAB member surveys, and related comments were reviewed during these sessions.

#### Triangulating results from different methods

We used a group-based reflection approach to triangulate qualitative and quantitative findings from the various sources to identify key lessons learned and strategies for documenting and assessing CAB member engagement.

## Results

### Documentation of CAB meeting processes

#### Attendance, primary language, and modality used

Attendance was high for both groups, with an average of 87% of CAB members present at each meeting. Attendance rates varied across subgroups, with Community Partners attending the most meetings (94% in CO-CREATE and 87% in STOP COVID-19 CA), Health Clinic Partners attending 91% of meetings, and Public Health Partners attending 75% of meetings. Most CAB members arrived on time and stayed for the entire duration of the meeting. The primary language was English across all subgroups in both projects except for the Community Members of the CO-CREATE CAB, where the primary language was Spanish. Participants predominantly attended meetings using a computer and integrated audio versus calling in to the meeting via phone.

#### Time spent speaking

For the CO-CREATE CAB meetings, Community Partners spoke for an average of 22 min (18% of total meeting time), followed by Health Clinic Partners speaking for 24 min (20% of total meeting time). Public Health Partners spoke the least compared to the other groups at 17 min (14% of total meeting time). We noticed an increase in contribution times for the subgroups depending on the meeting topic. Health Clinic Partners were noticeably more active during meetings focused on understanding key contributing factors that drive equitable COVID-19 testing, vaccination, and clinical trial participation. Public Health Partners were more active during meetings focused on identifying actions to increase equitable testing, vaccination, and clinical trial participation. Community Partners were the most active during the meetings focused on contributing factors, conditions required for success, and the identification of measures of success meetings. More specifics about the purpose of each meeting are described elsewhere [23].

For the STOP COVID-19 CAB meetings, the average number of minutes Community Partners spoke was 27 (30% of the total meeting time), and the duration increased as meetings progressed. CAB members were most active during meetings focused on identifying the conditions required to eliminate disparities in COVID-19 vaccinations, the identification of measures of success, and the final presentation of the Theory of Change for CAB review and consensus. Policy partners primarily took on an observer role during the Theory of Change process, which covered most of the CAB meetings we included in our ethnographic documentation. While their contributions during the main sessions were limited and time spent speaking was not documented, they provided insights during the end of CAB meeting reflections in support of community members’ viewpoints and expressing gratitude for the space to hear directly from the community.

For both projects, the Global ARC had the largest contribution, speaking on average 36 min (40% of total meeting time), as expected given that they facilitated the meetings.

#### Partner roles

For both projects, at least one documenter noted that CAB members provided input in all the meetings (100%), and identified priorities and participated in program design in almost all the meetings (range 88 − 100%). As expected, the partner roles surveys showed that the Global ARC and research team set the agenda and led or co-led most meetings (71-100%) (Table 2). The values in Table 2 represent ratings of roles from multiple documenters that in some cases had differing views on the roles. For example, a 25% value in the CO-CREATE Community Partners “No Active Role” field means that at least one of our multiple documenters indicated ‘no active role’ for Community Partners in 25% of the CAB meetings (n = 8 sessions).

**Table 2:** Results from CO-CREATE and STOP COVID-19 CA CAB meetings indicating the % of meetings in which each partner was reported as serving in each role

	No Active Role	Provided Input	Identified Priorities	Participated in Program Design	Set the Agenda	Led or Co-led Meeting
**CO-CREATE**						
Community Partners	25%	100%	88%	100%	25%	13%
Health Clinic Partners	50%	100%	88%	88%	25%	13%
Public Health Partners	25%	100%	88%	88%	38%	0%
Global ARC	0%	88%	100%	100%	100%	100%
UCSD Research team	88%	100%	88%	88%	100%	88%
**STOP COVID-19 CA**						
Community Partners	0%	100%	100%	100%	29%	14%
Policy Partners	20%	100%	100%	100%	29%	0%
Global ARC	0%	86%	86%	86%	100%	100%
UCSD Research team	86%	71%	57%	100%	71%	71%

#### Interruptions

Logistics emerged as a theme for interruptions and included audio delays, bandwidth issues with video, technical issues with breakout rooms, as well as notes about screen sharing and other logistical processes related to the virtual meetings (Table 3). Asking for clarifications and/or explanations about the meeting procedures and sharing unsolicited opinions or responding to questions asked of the group were the other themes that emerged for stakeholder interruptions.

**Table 3:** Thematic analysis of interruptions by CAB members from CO-CREATE and STOP COVID-19 CA CAB meetings

	CO-CREATE	STOP COVID-19 CA
Clarifications/Explanations	33%	54.6%
Responses/Opinions	34.3%	31.8%
Logistics	32.9%	13.6%

#### Type of interactions by CAB sub-groups

Interactions were recorded and coded as having a sender and at least one target 4,540 times across the fifteen meetings (Table 4). The most frequent interaction type was providing information (44% in both CO-CREATE and STOP), followed by responding (38% in CO-CREATE and 37% in STOP). CAB members participated as the senders of information in 34% of interactions (35% in CO-CREATE and 31% in STOP) and as targets of communication in 16% (17% in CO-CREATE and 13% in STOP). The entire group was the most common target for both projects (26% in CO-CREATE and 27% in STOP COVID-19 CA). The patterns of types of interactions were similar for both projects.

**Table 4:** Frequency of interaction types by CAB sub-group for CO-CREATE and STOP COVID-19 CA CAB meetings*

Type of Interaction						
	Providing Info	Seeking Info	Response	Summation	Other	Total
**CO-CREATE**						n (%)
*Sender*						
Community Partners	120	15	117	14	2	268 (10.8%)
Health Clinic Partners	162	8	149	23	2	344 (13.9%)
Public Health Research Partners	117	8	113	9	1	248 (10.0%)
Global ARC	62	60	25	45	16	208 (8.4%)
UCSD Research team	11	19	11	2	0	43 (1.7%)
*Target*						
Community Partners	43	14	32	8	1	98 (4.0%)
Health Clinic Partners	81	3	87	5	0	176 (7.1%)
Public Health Research Partners	74	7	74	0	0	155 (6.3%)
Global ARC	125	25	125	1	4	280 (11.3%)
UCSD Research team	4	6	9	0	1	20 (0.8%)
Entire group	290	56	199	76	12	633 (25.6%)
Total	1089 (44.0%)	221 (8.9%)	941 (38.1%)	183 (7.4%)	39 (1.6%)	2473
**STOP COVID-19 CA**						
*Sender*						
Community Partners	286	45	269	35	3	638 (30.9%)
Policy Partners	12	5	15	7	0	39 (1.9%)
Global ARC	99	55	60	23	7	244 (11.8%)
UCSD Research team	42	11	23	0	0	76 (3.7%)
*Target*						
Community Partners	113	30	127	1	3	274 (13.3%)
Policy Partners	9	0	8	1	0	18 (0.8%)
Global ARC	49	33	95	1	3	181 (8.8%)
UCSD Research team	14	8	12	0	0	34 (1.6%)
Entire group	278	52	154	75	4	563 (27.2%)
Total	902 (43.6%)	239 (11.6%)	763 (36.9%)	143 (6.9%)	20 (0.9%)	2067

*Not all members of each sub-group participated in all meetings.

* An interaction is counted numerous times in this table because each communication has a sender and a target and because targets can be more than one group.

#### Thematic analysis of interactions

Rapid thematic analysis of stakeholder interactions identified three main categories: Theory of Change, Other, and Meeting Logistics. The most frequently discussed topics in both projects included contributions to the Theory of Change creation and Meeting Logistics. Within the Theory of Change category, providing input about sorting/naming ideas and providing ideas in breakout rooms were the most common themes for CO-CREATE, and providing input about sorting/naming ideas and summarizing ideas were the most common themes for STOP. Within the Other category, end of meeting reflections were the most common for CO-CREATE, and sharing stories/positive thoughts of the day and recruitment or data collection discussions were the most common themes for STOP, Within the Meeting Logistics category, language translation and Zoom/Miro were the most common themes for CO-CREATE, and agenda review/roll call and Zoom/Miro were the most common themes for STOP (Table 5). Reflections were solicited at the end of most meetings to explore what topics were most impactful for CAB members from a given meeting. Most reflections were specific to the topics discussed during the meeting and explored potential action steps for the research team and other partners. Key topics included: reference to community, trust, gratitude, access to vaccines, access to resources & testing, structural racism, and providing effective and/or consistent messaging.

**Table 5:** Thematic analysis of stakeholder interactions in CO-CREATE and STOP COVID-19 CA CAB meetings

	CO-CREATE	STOP COVID-19 CA
Total interactions	n = 795	n = 691
**Theory of Change**	n (%)	n (%)
Providing ideas for ToC in breakout room	258	82
• *Community/ Faith Leaders/ Work force*	*42 (16.3)*	*27 (32.9)*
• *Policy/ Government*	*51 (19.8)*	*14(17.7)*
• *Cultural/ Language*	*33 (12.8)*	*19 (23.2)*
• *Communication/ Misinformation*	*29 (11.2)*	*20 (24.4)*
• *Accessibility*	*50 (19.4)*	*13 (15.9)*
• *Resources /Housing/ Employment/ Transport*	*29 (11.2)*	*0 (0)*
• *Vaccine*	*10 (3.9)*	*18 (22.0)*
• *Other*	*14 (5.4)*	*7 (8.5)*
Providing input about sorting, naming ideas	174	215
Instructions/clarification about ToC exercise	69	76
Summarizing ideas	68	95
Appreciative Inquiry data presentation and feedback	20	0
**Theory of Change - Total**	**649 (81.6)**	**471 (68.2)**
**Meeting logistics**	**n (%)**	**n (%)**
Language translation	24	7
Miro/Zoom	17	8
Other (connection issues, etc.)	12	4
Agenda review, roll call	11	8
Engagement surveys	3	7
Meeting schedule	4	4
Honorarium	2	4
Website	5	0
**Meeting logistics - Total**	**69 (8.7)**	**40 (5.8)**
**Other **	**n (%) **	**n (%)**
Reflections	53	44
Sharing stories, positive thought of day	**-**	40
Recruitment or data collection discussions	**-**	39
• *Language*	*-*	*13 (33.3)*
• *Methods of contact*	*-*	*13 (33.3)*
• *Sample*	*-*	*8 (20.5)*
• *Incentives*	*-*	*6 (15.4)*
Introductions	7	28
Background on COVID-19, project, ToC process	5	11
General questions about board, state of virus	3	8
Presentation to group with lit review, etc.	9	1
**Other - Total**	**77 (9.7)**	**180 (26.0)**

#### Refinement of documentation forms

Based on feedback shared by documenters about the usefulness of the sections of the documentation forms, multiple changes were made. Key modifications included removing eight items (e.g., late arrival, early departure), adding four new items (e.g., documentation method, time meeting started and ended), and modifying three items (e.g., added an option for using an interpreter, added location for each interaction such as main room, breakout room, etc.). The revised documentation form includes four key sections (Meeting, Actors, Acts, and Roles Survey). Sections on the revised form can be divided among documenters to reduce workload on any one documenter. The revised documentation form is provided in Supplemental Materials.

### Post-session survey of CAB members, the research team, and community partners on engagement

Response rates for the post-meeting survey were 76.5% for CO-CREATE CAB members and 73.9% for STOP CAB members. The quality and frequency of engagement was rated overall favorably. Almost all CAB members (98**–**100%) reported good or excellent engagement across domains for both projects. In the rare occasion when engagement was rated less favorably, it was more common within the STOP CAB by English-speaking community members. Table 6 shows the common themes that emerged when analyzing the open-ended comments provided at the end of the survey. Themes included gratitude and positive experiences related to the work the projects were doing (49% of comments for CO-CREATE and 44% for STOP), comments related to meeting engagement during virtual meetings (18% for CO-CREATE and 12% for STOP), input/suggestions to improve meeting processes (12% for CO-CREATE and 28% for STOP), input/suggestions about dissemination strategies (4% for both CO-CREATE and STOP), and thoughts about the impact of the CAB (2% for CO-CREATE and 8% for STOP). About 26% and 12% of comments were related specifically to interpretation or engagement of Spanish speaking board members for CO-CREATE and STOP, respectively.

**Table 6:** Thematic analysis of stakeholder engagement open-ended survey comments for CO-CREATE and STOP COVID-19 CA CAB meetings

	CO-CREATE	Example quotes	STOP COVID-19 CA	Example quotes
Gratitude	33%	Cada vez voy entendiendo mas este grupo gracias por toda la información que nos están compartiendo. Every time I am understanding this group more, thank you for all the information you share with us. *(Community Partner)* I am learning so much from this process. Thank you! *(Public Health Partner)*	8%	Les agradezco a toda esta agrupacion el esfuerzo que hacen para querer ayudar a nuestras comunidades mas vulnerables. Pero espero que se llegue a concretar algo y no solo ser parte de un estudio. Muchas gracias por tomar encuenta mi opinion. I thank all this group for the effort they put in to help our most vulnerable communities. But I do hope that something will come to fruition and not just be part of a study. Thank you very much for taking my opinion into account. *(Community Partner)*
Positive experience	16%	It’s a long process, but getting feedback from all the diverse voices actually leaves you with a good feeling at the end. *(Public Health Partner)* I was very impressed by the level of engagement from everyone in sorting through the measures. It was a new concept and process and I think everyone was resilient in transitioning. I also appreciated the genuine interest and questions about the evidence review presentation. *(non-CAB Research Partner)* This has been a great experience, love getting community input straight from them. *(Health Clinic Partner)*	36%	Me encanto todo li de la ultima ves ,me siento incluida esta todo el grupo participando gracias, Gracias cada ves estoy mas feliz y agradecida por toda esta ParticipaciÃ³n que tenemos, y trabajar todos I loved everything from the last time, I feel included the whole group is participating, thank you, Thank you every time I am more happy and grateful for all this participation that we have, and everyone works well together. *(Community Partner)* It is very nice to see our partners share their experiences and suggestions. I noticed increasing participation both in the larger group and in our small breakout groups. The process is very important and what we find is helpful. *(non-CAB Research Partner)*
Meeting engagement *--- (engagement related to Spanish speakers)*	18% *(10%)*	I value the efforts made to pivot and try and make the Spanish language collaborators more integrated into the full process. I can tell that it is evolving, and I value the work being done on this front. I say this because I realized my responses to the first few questions were really only reflective of the English-facing activities because that is the language I participate in. And I need to remember to not just jump in and talk right away, so I create that space for others. Another thought is calling for Spanish language responses first at least half of the time instead of pausing to ask at the end. None of this is a criticism - just a reflection of things I’m learning about how I can build in more responsiveness in my own work outside of this process. *(Public Health Partner)* Much better facilitation for our Spanish speaking partners - created better place for open discussion and didn’t feel as time pressured. Seeing the board can be a bit challenging. *(Health Clinic Partner)*	12% *(8%)*	It was nice seeing everyone (both English and Spanish speakers) actively engaged in the topic. It was good that the English speakers were actively aware of doing things like taking time to repeat phrases/provides the Spanish speakers time to speak. *(non-CAB Research Partner)*
Input - Meeting process	12%	Gracias por los breaks muy importante Thanks for the breaks, they’re very important *(Community Partner)* When reporting back it would be great to start with community members and not the public health researchers; this can help avoid (perceptions of) hierarchies based on academic training. *(Public Health Partner)* Thank you for including me in this meeting. Moving forward, I would like to hear more from the community board members regarding their own experiences with testing access and vaccines. thank you. *(Health Clinic Partner)*	28%	I really like the way we move slowly to reach our goal as a team. Many breakout rooms are really helpful to be inclusive. *(Community Partner)* Although the moderator makes every effort to give everyone the opportunity to weigh in, some members regularly dominate the discussion, which affects others’ ability to share their opinions and perspective. It might help on occasion to call on members, starting in the order of the ones we hear from the least. *(non-CAB Research Partner)*
Input - Interpretation	16%	Es acerca del sistema de traduccion.no estamos teniendo muy buen resultado.deberia ser en un solo electronico.asi evitariamos confuciones. Regarding the translation system … we are not having very good results … it should be in a single electronic … so we would avoid confusion. *(Community Partner)*	4%	A mi parecer ,todos sabemos lo que se tiene que tener en Nuestras Comunidad y lo expresamos de diferentes maneras. Hay palabras como ayer cuando se menciono creo que no es la palabra correcta, y como lo dije puedo no hablar, entender ,leer ingles pero es porque no es mi Lenguage Original. Pero soy un profecioal en mi pais Pero si me explican lo que acontece, o la cituacion que esta sucediendo en mi idioma ,claro Creando Confianza en la Comunidad In my opinion, we all know what we need in our community, and we express it in different ways. There are words like yesterday that were mentioned were incorrect, and as I said I cannot speak, understand, read English but it is because it is not my original language. But I am a professional in my country, but if they explain to me what is happening, or the situation that is happening in my language, of course creating trust in the community. *(Community Partner)*
Input - Dissemination	4%	En mi experiencia y recaudando la información de la comunidad la prueba para el covid está siendo olvidada por que ahora la vacuna es lo que consideran prioridad…así que las filas para el covid descienden y las de la vacuna aumenta esto más de ser un alivio es preocupante. Las vacunas no están tan disponibles y aunque se la pongan no salvan del covid solo disminuyen los efectos y esto es tan importante que trasmita para que la población no baje su guardia y continúen con las pruebas del covid.en resumen educación e información serteraaaa [certera] gracias por este espacio gracias. In my experience through collecting information from the community, the COVID-19 test is being forgotten because now the vaccine is what they consider a priority, so the lines for the covid test decrease and those for the vaccine increase; this more than being a relief, it is worrisome. Vaccines are not so available and even if they get it, they do not save us from covid they only reduce the effects and this is so important to communicate so that the population does not lower their guard and continue getting tested. In summary, education and accurate information. Thanks for this space, thank you. *(Community Partner)*	4%	The workshop is great way on how the community members share their thoughts on how their members react to the COVID19 crisis, it also help leaders to take messages back to their community on how to understand the benefits on how they can protect themselves with COVID19. *(Community Partner)*
CAB impact	2%	Judging by the comments at the conclusion of yesterday’s meeting, the Advisory Board process is already having a positive impact within San Ysidro Health. Some representatives from the SYH partners said that they will make some adjustments to their work right now, and we haven even finished the planning process. (*Non-CAB Community Partner)*	8%	The discussion benefits the partners where the community board will share this information from discussion with their community members. For example, the difficulty of the vaccine among the minority community where they have a hard time getting appointments. Also, community board members can help to fill the gap for language that makes confusion among the minority community about the vaccine, I hope many people now trust their community leaders and are willing to get the COVID19 vaccine. *(Community Partner)*

### Periodic reflections and Debriefing Sessions

Periodic reflections and debriefing sessions led to several methodological refinements for the documentation process and resulted in revised documentation forms. When less than perfect ratings or qualitative comments were identified from the stakeholder surveys, the research team and Global ARC team discussed their potential causes and developed strategies to address them for the next session. In addition, when concerns were detected during our after-meeting debrief sessions with the research team and Global ARC team, changes for the improvement of engagement in future sessions were discussed. Debriefing sessions with the documenters identified challenges related to technology issues and the ability to accurately document content. The virtual format of the meetings limited ability to document body language and behavioral nuances, particularly when meetings were documented using the Zoom recording because of the limited number of participants that show on the screen with the recording. We were not able to record in all breakout rooms, which resulted in some missing data. Pre-assigning documenters to focus on specific CAB sub-groups along with the ability to record CAB meetings for repeated review made documentation more feasible. All of these processes led to an iterative refinement of our CAB processes.

## Discussion

We report the development and application of a multi-method ethnographic approach to documenting and assessing community engagement in two virtual CABs focused on co-creating strategies for equitable COVID-19 testing, vaccination, and clinical trial participation for underserved communities. Assessing partner engagement through multiple methods allowed for nuanced ethnographic data collection that refined our local work with CAB members and contributes more broadly to the needed literature and pragmatic resources for evaluating community engagement in health implementation research. It is suggested that a key approach to assess meaningful engagement is to explore if participants feel empowered during the process and if the engagement results in change [27]. Goodman and Sanders Thompson [28] posed important questions when evaluating stakeholder engagement in research that our work aimed to address including: Which are the appropriate stakeholders to engage to address a problem?; Where is your partnership on the stakeholder engagement continuum?; What processes should be developed and used for partnership sustainability and progress along the stakeholder engagement continuum?; How will you evaluate the quality and quantity of stakeholder engagement?

A primary motivator for this report and accompanying documentation forms was the scarcity of methodological knowledge and dissemination of community engagement evaluation tools. Most available tools rely on surveys and self-assessment by group members (e.g., Healthy People, Coalition Self-Assessment [29]). These instruments are relatively lengthy and focus on self-reported information about the content and functioning of a coalition.

In our work, we expanded a pragmatic survey of the quality and extent of quality engagement adapted from Goodman and colleagues [25] with structured ethnographic documentation of the CAB sessions. Information from these two sources were used in real-time as part of the periodic reflections between the Global ARC and the research team to refine the structure and conduct of the proceeding CAB meetings. Specific modifications based on these data included selecting a standing day and time for CAB meetings to increase predictability; changing how feedback requests were structured during the CAB sessions (e.g., providing clear context, making requests specific); refining language support for non-English speakers (e.g., English speakers were asked to speak slower, support for non-English speaking members was provided on how to access interpretation); and addressing group/perceived power dynamics (e.g., encouraging CAB members who had less opportunity to contribute to share during meetings).

Overall, engagement was strong across CAB meetings, member types, and projects as demonstrated by multiple methods. At the foundation, attendance at the CAB meetings was high, and most CAB members stayed for the full meeting duration for both projects. When looking at time spent speaking, the average contribution of all CAB partners in the CO-CREATE project was close to 60%, and CAB members’ contributions increased over the course of the CAB convenings. Since the main focus of the CAB meetings are to learn from the CAB members, our team found these high CAB contribution levels encouraging.

An indicator of successful engagement of stakeholders in both projects was displayed in Table 2 in the form of partner roles. According to documenters, CAB members were engaged in diverse roles across the meetings, including providing input, identifying priorities, and participating in program design in 88-100% of meetings. While to a lesser degree, they also engaged in agenda-setting and led or co-led the CAB meetings (25–29% and 13–14% of meetings, respectively). These values represent ratings of roles from multiple documenters that had differing views in some cases. Ongoing reconciliation of role definitions across documenters is an important activity during debriefing sessions. Ensuring the engagement of CAB members in various active roles is a desirable strategy to achieve meaningful engagement. While roles naturally change as projects progress, it is important to continue considering opportunities to invite participation from community partners in more active roles. Policy partners played a less active role than community members during the Theory of Change process but shared valuable insights during end of meeting reflections. The reflections were noted as being supportive of and validating community members’ viewpoints on topics such as access to vaccines, structural racism, and community/health policy. Policy partners also expressed appreciation for having the space to hear directly from the community. We found that a key theme for interruptions included challenges with audio delays and bandwidth issues with video. Transition to a virtual platform for our CABs as a response to the COVID-19 pandemic generated several necessary adaptations and accommodations. While technology challenges observed and reported by CAB members decreased over time, there is a need to heavily research and pilot virtual platforms to reduce time spent on logistics during meeting times. To support participation from CAB members, our team provided Chromebooks and internet hotspots, as well as ongoing technical assistance to our CAB members, especially those representing communities. It is critical to budget for resources to support the engagement of CAB partners. In our companion paper, we describe a pragmatic method to assess resources needed for initial and ongoing stakeholder engagement [24].

A second key theme from analyzed interruptions reflected discussions about meeting logistics (23%). Our team found it critical to create clarity around the processes and rules of engagement in our CAB meetings. An agenda that followed a predictable and set structure allowed us to check in with CAB members about key logistical issues that were important to them, including plans for the session, technology access, honorarium payments, and the timing for the next meeting. Explicitly earmarking meeting time for addressing concerns from CAB members during each session reinforced our shared partnership and interest in bi-directional knowledge exchange.

Finally, our assessment of engagement using the post-CAB meeting survey based on Goodman and colleagues’ instrument indicated a high level of satisfaction with the extent and quality of engagement across all groups. Throughout our interactions with CAB members, we expected the roles and content of engagement to change over time to align with the needs and priorities of the project, but the quality and extent of engagement was consistently perceived as positive by the CAB members. A key adaptation for this data collection was to add an open-ended comment box to the end of the survey allowing for specific feedback from participants. These comments provided a rich source of data and allowed each CAB member the opportunity to share. This was particularly important for CAB members who were less comfortable sharing during group discussions. Our thematic analysis of open-ended comments included sharing of positive experiences; reflections of engagement especially of Spanish speaking CAB partners; gratitude; input regarding the meeting process, interpretation, and dissemination; and thoughts about the impact of the CAB on the CAB member individually or on their community. As an additional step for inclusion and sharing, we presented these results to the CO-CREATE CAB. The members of the CO-CREATE CAB endorsed the findings and expressed appreciation for being part of the group and that their perspectives were elevated throughout the process. We were unable to do a similar sharing with the STOP CAB because their work was completed before the analysis and this manuscript were ready. However, we plan to electronically share the manuscript with them when published.

## Conclusion

When undertaking the assessment of community engagement, it is desirable to take a multi-method, longitudinal approach where the quality and the extent of engagement is monitored over time using diverse perspectives and techniques. In our projects, we benefited from a team of undergraduate and Master’s-level research assistants who were trained to conduct documentation of the engagement process. The initial documentation form was found to be overly comprehensive, and not all sections were necessary for community-engaged research projects, especially for rapid response projects like our COVID-19 work. Thus, we revised and substantially simplified the structured documentation form based on systematic feedback from those utilizing the form in the first part of the project with the intention of creating a pragmatic process that can be used across projects. Research teams and their partners are encouraged to adapt the structured documentation form to align with the priorities and context of their specific projects (Supplementary Materials). Furthermore, we recommend ongoing trainings, including debriefing and periodic reflection sessions, and routinely assessing data to strengthen methods and processes for meaningful community engagement. If problems related to community engagement are identified, adjustments to activities can be made in real time to advance the project beyond what would have been possible without utilizing ethnographic approaches.

Our findings, and importantly, the ethnographic methods described in this paper have a great promise to inform and be applicable to other local and global public health implementation efforts. A motivation of this current work was to address a significant methodological and pragmatic gap related to robust community engagement assessment. We demonstrated one such methodological package that could be transferred to other community settings. Specifically, we suggest that all local and global community-engaged research projects incorporate some form of assessment of the quality and extent of engagement. Ideally, these assessments use a multi-method approach and are conducted iteratively with an opportunity to adjust engagement practices throughout the life course of the project to respond to data emerging from these assessments.

Our multi-method process described here and the accompanying ethnographic documentation form complement existing resources for engaging stakeholders in a meaningful way. Use of theories, models, and frameworks that guide the engagement of community partners have been compiled by Pinto and colleagues [3]. Innovative techniques that allow for the engagement of diverse stakeholders have been created by Kwan and colleagues in the form of a webtool named the Stakeholder Engagement Method Navigator [30]. This Navigator is a collection of diverse partner engagement tools and resources. Our manuscript describes one method to increase meaningful partner engagement that could eventually be included in the Navigator. Our methods and findings also extend recent published work from Jolles Perez and colleagues [31] and Casillas and colleagues [32] that showcase applications and principles for authentic community engagement in public health research.

While additional research and practice are needed, our work begins to address the limitations and opportunities highlighted by Esmail, Moore, and Rein [15] regarding greater availability of robust quantitative, qualitative, and mixed methods approaches to evaluating community engagement. While robust and multi-method, our work is limited because we were unable to examine the impact of our engagement methods on the public health outcomes of the two research projects that the virtual CABs supported. This is an important and unanswered question in the implementation science field about the quantity and direct impact of meaningful community engagement on the clinical and implementation outcomes of a public health campaign or program. The analysis presented in this paper was also unable to address all of the important questions informing partner engagement; however, the primary purpose of our manuscript was to describe a methodology for assessing partner engagement. Future studies can expand the assessment form with additional fields to explore questions such as “Which are the appropriate partners to engage to address a problem?” Despite these limitations, this study is one of the first to rigorously report a multi and mixed methods approach to documenting and evaluating community engagement in implementation science projects. This study highlights the potential of ethnographic methods to facilitate learning with and from community partners, evaluate community engagement in health research, and bridging the research to practice gap.

## Electronic supplementary material

Below is the link to the electronic supplementary material.

Supplementary Material 1

## Data Availability

The datasets generated and/or analysed during the current study are not publicly available due to the majority of the data being qualitative and ethnographic in nature, so there are restrictions to sharing to preserve the privacy of individuals. However, the co-author team will review data requests and that data will be made available as reasonably appropriate. Contact lead author for data requests at barabin@health.ucsd.edu.

## Abbreviations

CAB: Community Advisory Board
STOP COVID-19 CA: Share, Trust, Organize, Partner: The COVID-19 California Alliance
CO-CREATE: Community-driven Optimization of COVID-19 testing to Reach and Engage underserved Areas for Testing Equity
BIPOC: Black, Indigenous, and People of Color
CEAL: Community Engagement Alliance Against COVID-19 Disparities
RADx-UP: Rapid Acceleration of Diagnostics-Underserved Populations
