# Supplementary material for: Using ethnographic approaches to document, evaluate, and facilitate virtual community-engaged implementation research
Source: BMC Public Health. 2023 Feb 28;23:409. doi: 10.1186/s12889-023-15299-2 (PMC9974043; doi:10.1186/s12889-023-15299-2)
Supplement: Supplementary file 1 — Supplementary Material 1 [file 12889_2023_15299_MOESM1_ESM.pdf]

INNOVATION DOCUMENTATION FORM

SECTION 1: MEETING

Documenter: __________________________ **Meeting:** ___________________ **Date:** ____________________

Scene (e.g., main room, interpretation room, small groups, breakout rooms):

Technology (e.g., Zoom, Miro, white board):

Documentation method:

☐ **Live** ☐ **Recording** ☐ **Both**

Purpose/Agenda for the meeting:

Were all agenda items discussed? ☐ Yes ☐ **No**

If no, what was the reason for not addressing all items?

Time meeting started (note if meeting started late):

Time meeting ended (note if meeting ended early or late):

SECTION 2: ACTORS

Group (e.g., CAB Community members): (*repeat as needed for additional groups*)

☐ **Name, Organization, Partner role (*complete prior to meeting if possible*)**

Comments *(include information about whether person arrived late or left early and time if known, and if there were technology issues (unstable internet, phone connection issues) and whether this had an impact on the quality of communication or conversation)*:

____

____

Time talking (enter in number of minutes): ______ ______ ______ ______ ______ ______ ______ ______ ______ ______ ______

Primary language for participation: ______________________________ ☐ **Used interpreter**

Interrupts (Who, why): ____

☐ **Name, Organization, Partner role (*complete prior to meeting if possible; repeat as needed for additional members*)**

Comments *(include information about whether person arrived late or left early and time if known, and if there were technology issues (unstable internet, phone connection issues) and whether this had an impact on the quality of communication or conversation)*:

____

____

Time talking (enter in number of minutes): ______ ______ ______ ______ ______ ______ ______ ______ ______ ______ ______

Primary language for participation: ______________________________ ☐ **Used interpreter**

Interrupts (Who, why): ____

Is there variation in terms of engagement across language groups?

Additional observations not listed above:

CALCULATE AFTER MEETING:

**How much time did each group talk during the meeting?**<br><br>CAB Community members: ___________ total minutes<br><br>***Add totals for additional groups as needed***

SECTION 3: ACTS

**Sender**<br>**Who and What**	**Target**<br>**To whom: individual, sub-group, entire group**	**Scene**<br>**(select one)**	**Type**<br>**(select all that apply)**<br><br>**Seeking info:** Asking for information from individual/group<br>**Giving info:** Providing unsolicited facts, data, or opinion, providing information as a response<br>**Agreement:** Agreeing with or endorsing others statements or summaries<br>**Summation:** Summarizing points and making conclusions<br>**Closing**: closing statement at end of meeting	**Comments**
		☐ **Main room/Entire group**<br>☐ **Breakout room/Small group**<br>☐ **Chat**<br>☐ **Other:** _______________	☐ **Seeking Info**<br>☐ **Giving Info**<br>☐ **Agreement**<br>☐ **Summation**<br>☐ **Closing**<br>☐ **Other**	
		☐ **Main room/Entire group**<br>☐ **Breakout room/Small group**<br>☐ **Chat**<br>☐ **Other:** _______________	☐ **Seeking Info**<br>☐ **Giving Info**<br>☐ **Agreement**<br>☐ **Summation**<br>☐ **Closing**<br>☐ **Other**	
Add rows as needed<br>(average for our 2 hour CAB meetings was 92 Acts, range 10-177 Acts)				

**Additional/overall documentations and notes not listed above:**

SECTION 4: PARTNER SURVEY

Given what you have seen and heard in this meeting, how would you describe the role of each partner listed below in this meeting? (Select all that apply)

	No Active Role	Provided Input	Identified Priorities	Participated in Program Design	Set the Agenda	Led or co-led Meeting
**CAB Community members**	☐	☐	☐	☐	☐	☐
Add rows as needed for additional groups	☐	☐	☐	☐	☐	☐
